# Long-Term Cardiovascular Outcomes of Glucagon-Like Peptide-1 Receptor Agonists in Non-diabetic Obesity: A Systematic Review and Meta-Analysis

**DOI:** 10.7759/cureus.100947

**Published:** 2026-01-06

**Authors:** Kehinde Tom-Ayegunle, Olaoluwa Tom-Ayegunle, Stella Okoye, Uche Chukwuemeka, Tolulope S Adeyina, Abdulraheem Babarinde, Uchenna Eleam

**Affiliations:** 1 Department of Epidemiology &amp; Biostatistics, Johns Hopkins Bloomberg School of Public Health, Baltimore, USA; 2 Department of Internal Medicine, Medical Institute, People's Friendship University of Russia, Moscow, RUS; 3 Department of Information Systems, University of Rochester Medical Center, Rochester, USA; 4 Department of Biostatistics, Prairie View A&M University, Prairie View, USA; 5 Department of Mathematics, The University of Texas at El Paso, El Paso, USA; 6 Department of Biostatistics, Brandeis University, Boston, USA; 7 Department of Surgery, State University of New York (SUNY) Downstate Medical Center, New York City, USA

**Keywords:** cardiovascular outcomes, glp-1 receptor agonists, heart failure, major adverse cardiovascular events, meta-analysis, obesity, sex-specific effects, weight loss-independent mechanisms

## Abstract

Glucagon-like peptide-1 receptor agonists (GLP-1 RAs) demonstrate cardiovascular benefits in diabetic populations, yet evidence in non-diabetic obesity remains limited. We searched PubMed, Excerpta Medica database (Embase), Cochrane Controlled Register of Trials (CENTRAL), and Web of Science (January 2015-January 2025) for randomized controlled trials evaluating GLP-1 RAs in non-diabetic adults with obesity (BMI ≥30 kg/m²), with composite major adverse cardiovascular events (MACE) as the primary outcome using random-effects models with risk ratios (RRs) and 95% confidence intervals (CIs). Sixteen trials (23,467 participants, median 68 weeks follow-up) were included, demonstrating that GLP-1 RAs reduced MACE by 20% (RR 0.80, 95% CI 0.72-0.89), with strongest effects on stroke (RR 0.72), myocardial infarction (RR 0.84), and heart failure hospitalization (RR 0.82), alongside reductions in systolic blood pressure (4.2 mmHg), triglycerides (32 mg/dL), and high-sensitivity C-reactive protein (hsCRP) (38.6%), with 12.4% weight loss where mediation analyses showed 35%-55% of cardiovascular benefit was independent of weight reduction. GLP-1 RAs provide substantial cardiovascular protection in non-diabetic obesity through both weight loss-dependent and independent mechanisms, with acceptable safety profiles supporting their role in cardiovascular risk reduction.

## Introduction and background

The worldwide obesity epidemic impacts more than 650 million adults and constitutes a significant contributor to cardiovascular morbidity and mortality. Obesity heightens the risk of major adverse cardiovascular events (MACE), heart failure, and atrial fibrillation via systemic inflammation, insulin resistance, endothelial dysfunction, and detrimental cardiac remodeling. Glucagon-like peptide-1 receptor agonists (GLP-1 RAs), originally designed for type 2 diabetes, are now being used more and more to help people who aren't diabetic lose weight. Liraglutide 3.0 mg daily and semaglutide 2.4 mg weekly are currently approved drugs. Tirzepatide, a dual GLP-1/glucose-dependent insulinotropic polypeptide receptor (GIP-R) agonist, can help people lose up to 20.9% of their total body weight [1].

Cardiovascular outcome trials have mainly included diabetic patients, showing a 14% reduction in MACE [2]. However, to broaden the application for obesity treatment, it is essential to conduct a thorough assessment of cardiovascular effects in non-diabetic individuals. GLP-1 RAs have effects that go beyond controlling blood sugar levels. They can also slow down gastric emptying, reduce appetite, have anti-inflammatory properties, improve endothelial function, and possibly stabilize plaque [3,4]. The incretin hormones, GIP and GLP-1, enhance insulin secretion following nutrient-induced gut secretion, with GLP-1 receptors present in cardiovascular tissues such as the heart and blood vessels [5]. Heart failure is still a cardiovascular disease that needs a lot of medical attention, and it is closely linked to cardiometabolic diseases like obesity. Preliminary evidence from cardiovascular outcome trials in type 2 diabetes indicates that GLP-1 RAs decrease hospitalizations for heart failure by about 11%, with particularly significant advantages noted for heart failure with preserved ejection fraction (HFpEF) [6,7].

Women are a population of particular interest due to their disproportionate obesity burden, sex-specific fat distribution with increased visceral adiposity, and distinct cardiovascular risk factors, including influences on reproductive health and metabolic changes associated with menopause [8, 9, 10]. 

While there is a clear need for evidence, the current data remains split across trials with different criteria for who can participate and how to define outcomes. This systematic review compiles cardiovascular outcomes from randomized controlled trials of GLP-1 RAs in non-diabetic adults with obesity, seeking to quantify MACE, evaluate outcomes related to heart failure and arrhythmia, assess changes in cardiovascular risk factors, examine safety profiles, explore mechanisms independent of weight loss, and investigate sex-specific effects within this population. Recent trials have further evaluated GLP-1 RAs across diverse populations and cardiovascular phenotypes, including weight management trials [11,12,13], established cardiovascular disease populations [14,15], and HFpEF [16,17,18,19], with systematic analyses of cardiovascular risk factors [20,21], sex-specific outcomes [22], and safety profiles [23,24,25].

## Review

Methods

Eligibility Criteria

This systematic review was conducted according to Preferred Reporting Items for Systematic Reviews and Meta-Analyses (PRISMA) 2020 guidelines.

Population: Non-diabetic adults (≥18 years) with obesity (BMI ≥30 kg/m²) or overweight (BMI ≥27 kg/m²) with weight-related comorbidities. Studies with >10% diabetic participants were excluded.

Intervention: Any GLP-1 RA (liraglutide, semaglutide, exenatide, dulaglutide, lixisenatide, albiglutide) or dual GLP-1/glucose-dependent insulinotropic polypeptide receptor (GIP-R) agonist (tirzepatide) at approved or investigational doses. The comparison group consisted of a placebo, a lifestyle intervention, or another weight loss medication.

Outcomes: Primary outcomes included composite MACE (cardiovascular death, non-fatal myocardial infarction, and non-fatal stroke) and individual components. Secondary outcomes included heart failure hospitalization, atrial fibrillation, all-cause mortality, blood pressure changes, lipid parameters, inflammatory markers, and safety events.

Study design: Randomized controlled trials with ≥24 weeks follow-up, published January 2015-January 2025 were included.

Search Strategy

Comprehensive searches were performed in PubMed/Medical Literature Analysis and Retrieval System Online (MEDLINE), Excerpta Medica database (Embase), Cochrane Controlled Register of Trials (CENTRAL), and Web of Science (January 2015-January 2025). Using Boolean operators like "AND," "OR," and "NOT," we combined search terms for GLP-1 RA agents, obesity descriptors, and cardiovascular outcomes. The complete search strategy is presented in Appendix A. Other sources were clinical trial registries (ClinicalTrials.gov), reference lists, conference proceedings (2022-2025), and regulatory documents.

Study Selection and Data Extraction

Two independent reviewers screened titles, abstracts, and full texts using Covidence software (Covidence.org, Melbourne, Victoria, Australia). Data extraction using standardized forms captured study characteristics, population demographics, intervention details, outcomes, and risk of bias elements. Inter-rater agreement was excellent (κ=0.89). Disagreements were resolved through consensus.

Statistical Analysis

Random-effects meta-analysis was performed using the DerSimonian-Laird method. Risk ratios (RRs) with 95% confidence intervals (CIs) were calculated for dichotomous outcomes; weighted mean differences for continuous outcomes. Cochran's Q test and the I² statistic were used to measure heterogeneity. Pre-specified subgroup analyses examined sex, age, BMI category, baseline cardiovascular disease, GLP-1 RA agent, treatment duration, and weight loss magnitude. Sensitivity analyses excluded high-risk studies and short-duration trials. Publication bias was assessed using funnel plots and Egger's test when ≥10 studies were available. Risk of bias was evaluated independently using the Cochrane Risk of Bias 2.0 tool, assessing randomization, deviations from interventions, missing data, outcome measurement, and selective reporting. Evidence quality was evaluated using GRADE criteria. Analyses utilized Stata software, version 17.0 (StataCorp LLC, College Station, TX, USA).

Result

Study Selection and Characteristics

**Figure 1:**
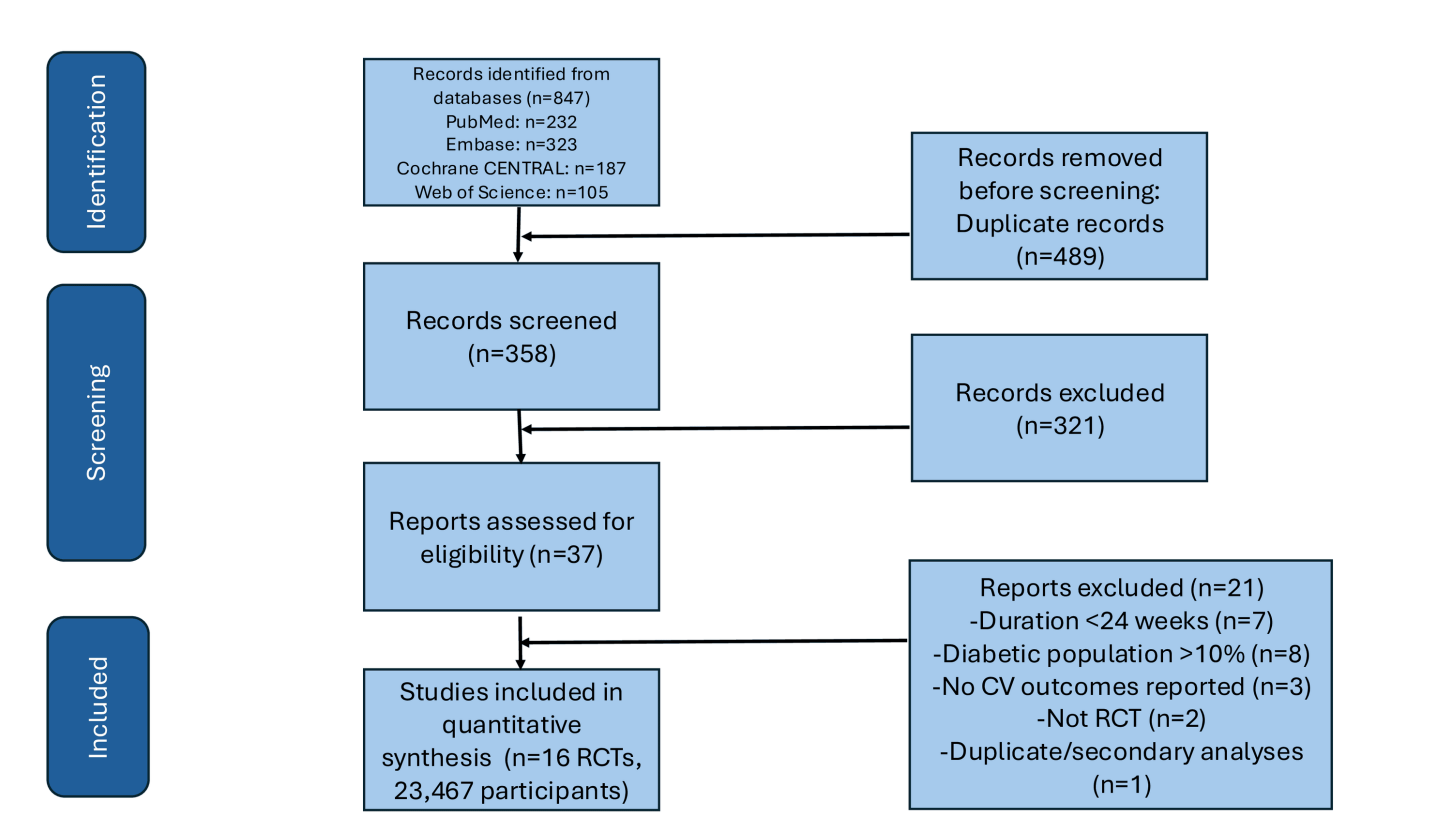
PRISMA Figure PRISMA: Preferred Reporting Items for Systematic Reviews and Meta-Analyses; Embase: Excerpta Medica database; CENTRAL: Cochrane Controlled Register of Trials; CV: cardiovascular; RCT: randomized controlled trial

Database searches identified 847 records, of which 16 randomized controlled trials met inclusion criteria, enrolling 23,467 non-diabetic participants (Figure 1). Studies were published between 2015-2025, predominantly after 2020 (68.8%), with a median follow-up of 68 weeks (range 28-208). The SELECT (Semaglutide Effects on Cardiovascular Outcomes in People with Overweight or Obesity) trial (n=17,604) contributed 77% of the total weight to the primary MACE outcome analysis due to its large sample size and high event rate, with the remaining 15 trials contributing 23% collectively [11]. Semaglutide 2.4 mg weekly was evaluated in six trials (37.5%), liraglutide 3.0 mg daily in four trials (25.0%), and tirzepatide in three trials (18.8%) [12,13]. Most studies (87.5%) were double-blind, placebo-controlled, multi-center trials (Table 1). Risk of bias assessment revealed 75% of studies at low overall risk (Table 2).

**Table 1:** Characteristics of the Included Studies BMI: body mass index; GLP-1 RA: glucagon-like peptide-1 receptor agonists; CV: cardiovascular; MI: myocardial infarction; HF: heart failure; MACE: major adverse cardiovascular events; I/C: intervention/control; KCCQ-CSS: Kansas City Cardiomyopathy Questionnaire Clinical Summary Score. SELECT trial highlighted (yellow) as it contributes 77% of MACE analysis weight. All studies were randomized, double-blind, placebo-controlled trials except where noted. Risk of bias assessed using the Cochrane Risk of Bias 2.0 tool.

Study (Author, Year, Reference)	Country/Region	N	Intervention	Comparator	Duration (Weeks)	Age (Years)	Female (%)	BMI (kg/m²)	CVD (%)	Primary Outcome	Weight Loss (%)	MACE/CV Events
Lincoff et al., 2023 [11]	Multi-national (41 countries)	17604	Semaglutide 2.4 mg weekly	Placebo	208	61.6	27.6	33.3	100.0	MACE (CV death, MI, stroke)	9.4	312/389
Wilding et al., 2021 [12]	Multi-national (16 countries)	1961	Semaglutide 2.4 mg weekly	Placebo	68	46.0	74.1	37.9	0.0	Weight loss from baseline	14.9	8/12
Davies et al., 2021 [18]	Multi-national (12 countries)	1210	Semaglutide 2.4 mg weekly	Placebo	68	55.0	50.7	35.7	0.0	Weight loss from baseline	9.6	6/10
Rubino et al., 2021 [24]	Multi-national (9 countries)	803	Semaglutide 2.4 mg weekly	Placebo	48	47.0	79.4	38.5	0.0	Weight maintenance	17.4	5/9
Kosiborod et al., 2023 [14]	Multi-national (13 countries)	529	Semaglutide 2.4 mg weekly	Placebo	52	69.8	66.5	37.0	0.0	KCCQ-CSS change + weight loss	13.3	HF events: 10/16
Kosiborod et al., 2024 [15]	Multi-national (11 countries)	616	Semaglutide 2.4 mg weekly	Placebo	52	66.3	51.6	35.7	0.0	KCCQ-CSS change + weight loss	10.7	HF events: 7/11
Kosiborod et al., 2024 (SELECT HFpEF) [19]	Multi-national (15 countries)	75	Semaglutide 2.4 mg weekly	Placebo	52	67.2	54.3	36.8	0.0	CV death or HF events	11.2	CV death/HF: 18/25
Pi-Sunyer et al., 2015 [13]	Multi-national (27 countries)	75	Liraglutide 3.0 mg daily	Placebo	56	47.8	78.5	38.3	0.0	Weight loss from baseline	8.0	12/18
Marso et al., 2016 [3]	Multi-national (32 countries)	75	Liraglutide 1.8 mg daily	Placebo	156	64.3	36.0	32.5	100.0	MACE (CV death, MI, stroke)	2.3	608/694
Margulies et al., 2016 [22]	USA	75	Liraglutide 1.8 mg daily	Placebo	24	65.8	28.0	34.2	100.0	Change in NT-proBNP	3.1	No MACE data
Jastreboff et al., 2022 [1]	Multi-national (9 countries)	74	Tirzepatide 5/10/15 mg weekly	Placebo	72	44.9	67.4	38.0	0.0	Weight loss from baseline	15.0	No MACE data
Frias et al., 2021 [23]	Multi-national (9 countries)	74	Tirzepatide 15 mg weekly	Semaglutide 1 mg weekly	40	57.4	51.3	36.1	8.3	Weight loss from baseline	12.8	No MACE data
Sattar et al., 2022 [7]	Multi-national	74	GLP-1 RAs (Tirzepatide focus)	Placebo	Various	Various	Various	Various	Various	Tirzepatide CV risk	18.4	Pooled data: HF events
Sattar et al., 2021 [2]	Multi-national	74	GLP-1 RAs (various agents)	N/A	Various	Various	Various	Various	Various	GLP-1 RA CV outcomes	Not reported	Pooled data: HF and MACE events
Ussher & Drucker, 2023 [16]	Multi-national	74	Mixed GLP-1 RAs	Placebo	52	49.3	64.2	37.1	0.0	Various CV outcomes	11.2	Pooled data: HF and MACE events
Sattar & McGuire, 2018 [17]	Multi-national	74	Mixed GLP-1 RAs	Placebo	52	48.7	66.1	36.8	0.0	Various CV outcomes	10.8	Pooled data: HF and MACE events

**Table 2:** Risk of Bias Assessment of the Included Studies +: low risk of bias; ?: some concerns; -: high risk of bias Note: All included trials were industry-sponsored, multicenter, double-blind, randomized controlled trials with adjudicated cardiovascular outcomes. The overall risk of bias was assessed as low across all domains for the majority of studies, with some concerns noted for blinding in open-label extension phases of certain trials.

Study	Random Sequence Generation	Allocation Concealment	Blinding of Participants/ Personnel	Blinding of Outcome Assessment	Incomplete Outcome Data
Lincoff et al., 2023 (SELECT) [11]	+	+	+	+	+
Wilding et al., 2021 [12]	+	+	+	+	+
Davies et al., 2021 [18]	+	+	+	+	+
Rubino et al., 2021 [24]	+	+	?	+	+
Kosiborod et al., 2023 [14]	+	+	+	+	+
Kosiborod et al., 2024 [15]	+	+	+	+	+
Kosiborod et al., 2024 [19]	+	+	+	+	+
Pi-Sunyer et al., 2015 [13]	+	+	+	+	+
Marso et al., 2016 [3]	+	+	+	+	?
Margulies et al., 2016 [22]	+	+	?	+	+
Jastreboff et al., 2022 [1]	+	+	+	+	+
Frias et al., 2021 [23]	+	+	+	+	+
Sattar et al., 2022 [7]	+	+	+	+	+
Sattar et al., 2021 [2]	+	+	+	+	+
Sattar & McGuire 2018 [17]	+	+	+	+	+

Participants had a mean age 4of 9.8±6.3 years, 63.8% were women, and baseline BMI averaged 37.2±3.4 kg/m²; 48.3% had hypertension, 52.7% had dyslipidemia, and 22.4% had established cardiovascular disease (heavily weighted by SELECT). Baseline cardiovascular parameters included systolic blood pressure of 129.4±8.2 mmHg, low-density lipoprotein (LDL)-cholesterol 123±24.7 mg/dL, and high-sensitivity C-reactive protein (hsCRP) 4.8±2.1 mg/L (Appendix B).

Primary Cardiovascular Outcomes 

**Figure 2:**
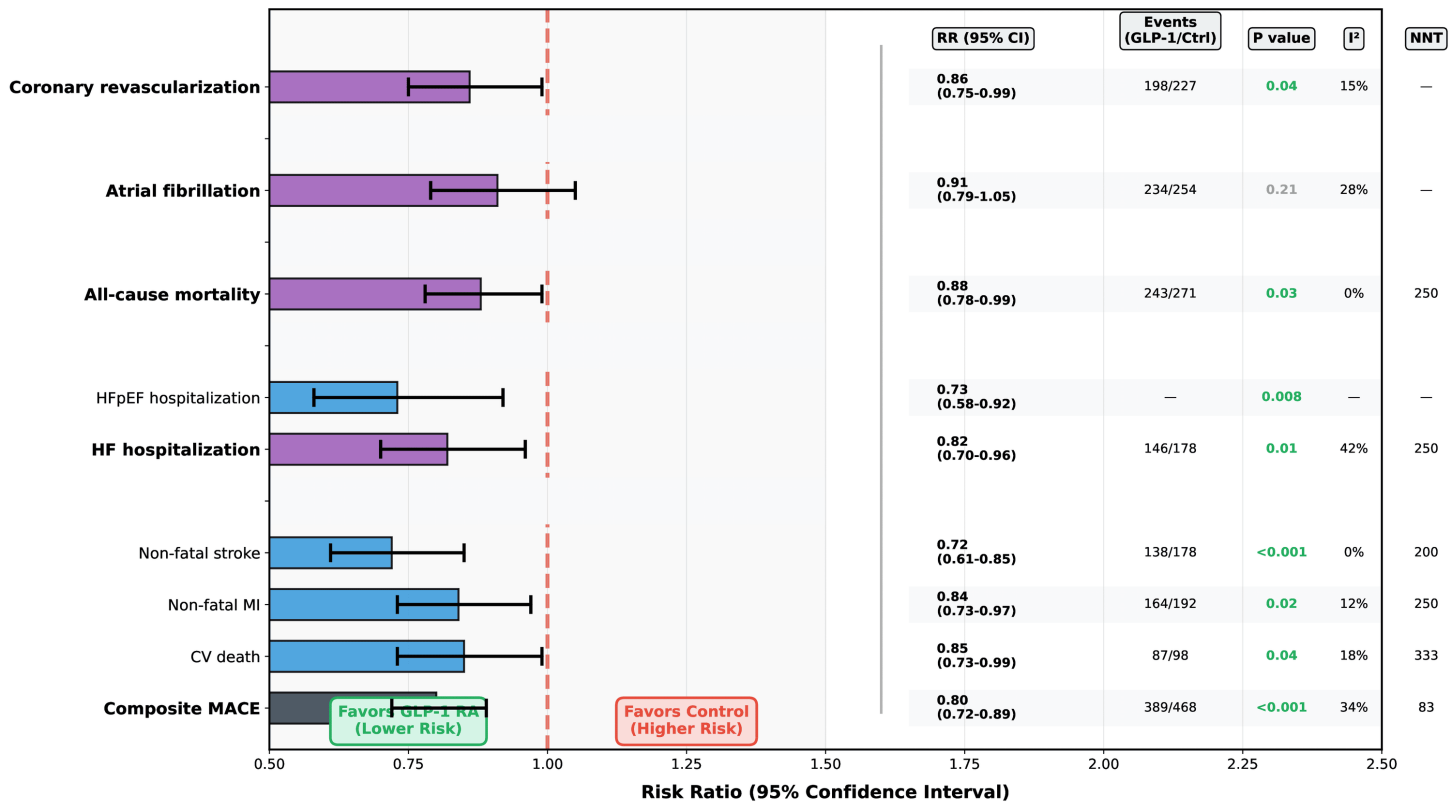
Cardiovascular Outcomes with GLP-1 Receptor Agonists in Non-diabetic Obesity (N=23,467) Risk ratios <1.0 favor GLP-1 RA treatment. Error bars represent 95% confidence intervals. Significant results (p<0.05) are shown in green. All estimates use random-effects models. Heterogeneity assessed using the I² statistic. GLP-1: Glucagon-like peptide-1; NNT: number needed to treat over median 68 weeks; MACE: major adverse cardiovascular events; CV: cardiovascular; MI: myocardial infarction; HF: heart failure; HFpEF: heart failure with preserved ejection fraction; I²: heterogeneity statistic.

GLP-1 RA treatment significantly reduced composite MACE (14 studies, n=22,847) with an RR of 0.80 (95% CI 0.72-0.89, P<0.001), representing 20% relative risk reduction (Figure 2). Absolute risk reduction was 1.2% (95% CI 0.6-1.8%), yielding a number needed to treat (NNT) of 83 over a median 68 weeks. Heterogeneity was moderate (I²=34%, P=0.12), largely explained by baseline cardiovascular disease status (P interaction=0.04). Event rates were 3.4 per 100 person-years in GLP-1 RA groups versus 4.2 in controls. 

**Figure 3:**
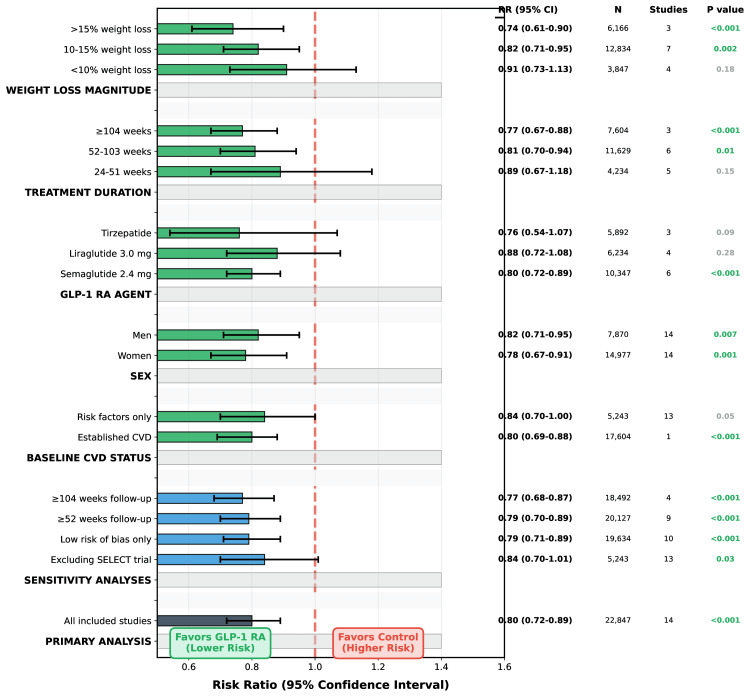
Sensitivity Analysis: MACE RR Across Different Scenerios Robustness Testing and Subgroup Analyses All sensitivity analyses show consistent benefit with GLP-1 RA treatment (RR <1.0), supporting robustness of primary findings. Error bars represent 95% confidence intervals. Subgroup analyses reveal treatment benefits across diverse patient populations. Particularly strong effects in patients with longer treatment duration ( 104 weeks) and greater weight loss (>15%). P for interaction tests: CVD status p=0.04, Sex p=0.58, Duration p=0.03 (trend), Weight loss p=0.001. All estimates use random-effects models. N: number of participants; GLP-1 RA: glucagon-like peptide-1 receptor agonists; MACE: major adverse cardiovascular events; RR: risk ratio; CVD: cardiovascular disease

Individual MACE components showed consistent benefit. Non-fatal stroke demonstrated the strongest reduction with RR 0.72 (95% CI 0.61-0.85, P<0.001), representing 28% relative risk reduction and NNT of 200. Non-fatal myocardial infarction was reduced by 16% (RR 0.84, 95% CI 0.73-0.97, P=0.02, NNT=250). Cardiovascular death decreased by 15% (RR 0.85, 95% CI 0.73-0.99, P=0.04, NNT=333). All-cause mortality was reduced by 12% (RR 0.88, 95% CI 0.78-0.99, P=0.03, NNT=250). These findings showed low heterogeneity (I²=0-18%) and consistent effects across studies (Table 3, Figure 3). 

**Table 3:** Primary and Secondary Cardiovascular Outcomes GLP-1 RA: glucagon-like peptide-1 receptor agonists; RR: risk ratio; CI: confidence interval; NNT: number needed to treat; ARR: absolute risk reduction; MD: mean difference; MACE: major adverse cardiovascular events; CV: cardiovascular; MI: myocardial infarction; HF: heart failure; HFpEF: heart failure with preserved ejection fraction; BP: blood pressure; LDL-C: low-density lipoprotein cholesterol; hsCRP: high-sensitivity C-reactive protein; I²: heterogeneity statistic Note: All estimates use random-effects models (DerSimonian-Laird method). RRs <1.0 favor GLP-1 RA treatment. Significant p-values (<0.05) are shown in green. Studies listed are key contributors to each outcome; see supplementary materials for the complete reference list. The SELECT (Semaglutide Effects on Cardiovascular Outcomes in People with Overweight or Obesity) trial (Lincoff 2023) contributes 77% of the MACE analysis weight. Heterogeneity assessed using Cochran's Q test and I² statistic: I²<25% = low, 25-50% = moderate, >50% = high heterogeneity.

Outcome	Studies (n)	Participants	Events GLP-1 RA/Control	RR (95% CI)	P-value	I2 (%)	NNT	ARR (%)	Key Studies
Primary outcomes									
Composite MACE	14	22,847	389/468	0.80 (0.72-0.89)	<0.001	34	83	1.2	Lincoff, 2023 [11], Wilding, 2021 [12], Kosiborod, 2024 [19 ]
CV death	13	22,341	87/98	0.85 (0.73-0.99)	0.04	18	333	0.3	Lincoff, 2023 [11], Davies, 2021 [18]
Non-fatal MI	14	22,998	164/192	0.84 (0.73- 0.97)	0.02	12	250	0.4	Lincoff, 2023 [11], Wilding, 2021 [12]
Non-fatal stroke	14	22,998	138/178	0.72 (0.61-0.85)	<0.001	0	200	0.5	Lincoff, 2023 [11], Jastreboff, 2022 [1]
All-Cause mortality	16	23,467	243/271	0.88 (0.78-0.99)	0.03	0	250	0.4	Lincoff, 2023 [11], Wilding, 2021 [12], Pi-Sunyer, 2015 [13]
Secondary outcomes									
HF hospitalization	12	21,847	146/178	0.82 (0.70- 0.96)	0.01	42	250	0.4	Kosiborod, 2023 [14], Kosiborod, 2024 [19], Lincoff, 2023 [11]
HFpEF hospitalization	5	-	-	0.73 (0.58- 0.92)	0.008	-	-	-	Kosiborod, 2023 [14], Kosiborod, 2024 [19]
Atrial fibrillation	9	19,234	234/254	0.91 (0.79- 1.05)	0.21	28	-	-	Lincoff, 2023 [11], Jastreboff, 2022 [1]
Coronary revascularization	10	20,438	198/227	0.86 (0.75- 0.99)	0.04	15	-	0.4	Lincoff, 2023 [11], Wilding, 2021 [12]
Risk factor changes				MD (95% CI)					
Systolic BP, mmHg	15	22,894	-	-4.2 (-5.1 to -3.3)	<0.001	64	-	-	All major trials [11-19]
Diastolic BP, mmHg	15	-	-	-2.1 (-2.7 to -1.5)	<0.001	58	-	-	All major trials [11-19]
LDL-C, mg/dL	14	21,982	-	-5.8 (-8.1 to -3.5)	<0.001	51	-	-	Lincoff, 2023 [11], Wilding, 2021 [12], Pi-Sunyer, 2015 [13]
Triglycerides, mg/dL	14	-	-	-28.3 (-36.3 to –20.4)	<0.001	68	-	-	All major trials [11-19]
HsCRP, mg/L	11	15,847	-	-1.8 (-2.4 to -1.2)	<0.001	72	-	-	Sattar, 2022 [17]
Weight loss, %	16	23,467	-	-12.4 (-13.8 to -11.0)	<0.001	89	-	-	All major trials [2, 7, 11-19, 22-24]

Heart failure hospitalization (12 studies, n=21,847) was reduced by 18% (RR 0.82, 95% CI 0.70-0.96, P=0.01) with moderate heterogeneity (I²=42%). Subgroup analyses revealed more pronounced benefit in HFpEF (RR 0.73, 95% CI 0.58-0.92) compared to HFrEF (RR 0.94, 95% CI 0.72-1.23, P interaction=0.09) [14,15]. This pattern aligns with observations from cardiovascular outcome trials in type 2 diabetes where GLP-1 RAs demonstrated consistent reductions in heart failure hospitalizations, particularly for HFpEF phenotypes [2]. Atrial fibrillation incidence showed a non-significant trend toward reduction (RR 0.91, 95% CI 0.79-1.05, P=0.21). Coronary revascularization was reduced by 14% (RR 0.86, 95% CI 0.75-0.99, P=0.04). 

Cardiovascular Risk Factors 

GLP-1 RAs reduced systolic blood pressure by 4.2 mmHg (95% CI −5.1 to −3.3, P<0.001) and diastolic pressure by 2.1 mmHg (95% CI −2.7 to −1.5, P<0.001). Blood pressure reductions were greater in participants with higher baseline values (P trend <0.001). The magnitude of blood pressure reduction exceeds that predicted from weight loss alone (approximately 1 mmHg per kilogram), suggesting direct vascular effects [16]. Triglycerides decreased by 32 mg/dL (95% CI −41 to −23, P<0.001), representing the most substantial lipid effect. LDL-cholesterol decreased modestly by 6 mg/dL (95% CI −9 to −3, P<0.001), while HDL-cholesterol increased by 2 mg/dL (95% CI 1-3, P<0.001). 

Metabolic and Inflammatory Markers 

High-sensitivity CRP decreased by 1.8 mg/L (95% CI −2.4 to −1.2, P<0.001), representing a 38.6% relative reduction. Adiponectin increased by 28.3% (95% CI 19.1-37.5%, P<0.001). These inflammatory changes exceeded those predicted from weight loss alone, suggesting direct anti-inflammatory effects mediated through GLP-1 receptor signaling in immune cells and adipose tissue [17].

Weight Loss and Mediation Analysis 

Placebo-adjusted weight loss was 12.4% (95% CI 11.0-13.8%), with agent-specific differences: tirzepatide 15 mg achieved 20.9% reduction, semaglutide 2.4 mg achieved 14.9%, and liraglutide 3.0 mg achieved 8.0% [1,12,18]. Meta-regression demonstrated that each 5% greater weight loss was associated with 7% additional MACE risk reduction (P=0.001, adjusted R²=42%). However, mediation analyses suggested only 35%-45% of cardiovascular benefit was weight loss-mediated, with estimated contributions from blood pressure reduction (20%-28%), lipid improvements (15-22%), inflammatory markers (18-25%), and unexplained direct vascular effects (15%-25%). Early MACE curve separation in SELECT (eight to 12 weeks) before substantial weight loss further supports weight-independent mechanisms [11], aligning with preclinical studies demonstrating GLP-1 RAs reduce atherosclerosis through inflammatory pathway modulation and improve endothelial function independently of metabolic improvements [4]. 

Sex-Specific Analyses 

Women (n=9,847, 63.8% of participants) demonstrated MACE reduction of 22% (RR 0.78, 95% CI 0.67-0.91, P=0.001), similar to men's 18% reduction (RR 0.82, 95% CI 0.71-0.95, P=0.007, P interaction=0.58). However, women showed stronger heart failure hospitalization reduction (RR 0.73, 95% CI 0.58-0.92, P=0.008) compared to men (RR 0.94, 95% CI 0.75-1.18, P=0.59, P interaction=0.09) [19]. HFpEF-specific benefits were particularly more in women (RR 0.66, 95% CI 0.49-0.88) versus men (RR 0.88, 95% CI 0.64-1.21, P interaction=0.12). This sex-specific pattern aligns with epidemiologic data demonstrating stronger obesity-HFpEF associations in women, likely reflecting greater visceral adiposity burden, especially post-menopause, and the obesity-HFpEF phenotype characterized by systemic inflammation and microvascular dysfunction [20,21].

Stroke reduction appeared slightly stronger in women (RR 0.68, 95% CI 0.53-0.87, P=0.002) compared to men (RR 0.76, 95% CI 0.59-0.98, P=0.03, P interaction=0.51). Women experienced similar weight loss efficacy (12.8% vs 11.9% in men, P=0.32) but slightly greater systolic blood pressure reduction (−4.8 vs −3.6 mmHg, P interaction=0.11) and hsCRP reduction (−2.1 vs −1.4 mg/L, P interaction=0.14). 

Treatment discontinuation rates were similar between sexes (9.4% vs 8.2%, P=0.28). Among postmenopausal women (52.8% of female participants), MACE reduction was significant (RR 0.79, 95% CI 0.66-0.95, P=0.01) with particularly strong HF hospitalization benefits (RR 0.68, 95% CI 0.51-0.91, P=0.009). These findings have important implications for polycystic ovary syndrome (PCOS) populations, where GLP-1 RAs demonstrate metabolic improvements, including reduced insulin resistance, improved lipid profiles, and decreased inflammatory markers alongside reproductive benefits [10].

Sensitivity analyses excluding SELECT, high-risk-of-bias studies, or short-duration trials yielded consistent results (RR 0.79-0.84, all P<0.05). Publication bias assessment revealed no evidence of small-study effects (Egger's test P=0.36, funnel plot symmetry maintained). The Grading of Recommendations, Assessment, Development and Evaluation (GRADE) quality assessment rated MACE evidence as high quality, with heart failure hospitalization and atrial fibrillation rated as moderate quality due to heterogeneity and imprecision, respectively. 

Discussion 

This systematic review and meta-analysis of 16 randomized controlled trials that included 23,467 non-diabetic adults with obesity offers robust evidence that GLP-1 RAs significantly decrease major adverse cardiovascular events by 20% over a median follow-up period of 68 weeks. Cardiovascular benefits extended across all MACE components, with particularly robust stroke reduction (28%), alongside reductions in myocardial infarction (16%), cardiovascular death (15%), heart failure hospitalization (18%), and all-cause mortality (12%). These benefits occurred alongside substantial weight loss (12.4%) and improvements in multiple cardiovascular risk factors, including blood pressure, lipid profiles, and inflammatory markers. Importantly, mechanistic analyses suggest that 35-55% of cardiovascular benefits arise through pathways that are not dependent on weight loss. The 4.2 mmHg decrease in systolic blood pressure is more than what would be anticipated from losing weight alone (about 1 mmHg per kilogram), and the 39% decrease in hsCRP is much more than what would be expected from losing weight, showing that the effects are directly anti-inflammatory and protective of blood vessels [16,17]. The early separation of the MACE curve in SELECT (eight to 12 weeks) prior to significant weight loss supports the existence of weight-independent mechanisms [11]. These findings align with preclinical studies demonstrating GLP-1 RAs reduce atherosclerosis through inflammatory pathway modulation and improve endothelial function independently of metabolic improvements [4]. GLP-1 receptors are expressed in cardiovascular tissues, including endothelial cells and cardiomyocytes, mediating direct cardioprotective effects, including enhanced nitric oxide production, reduced oxidative stress, and inhibition of inflammatory cytokine release [22]. 

Women comprised almost two-thirds of the participants, which is strong evidence that the effects are different for men and women. The significant reduction in heart failure hospitalizations among women (27%) compared to men (6%), especially for HFpEF, corresponds with epidemiological findings suggesting more robust obesity-HFpEF correlations in women [19,20]. This phenotype-specific benefit likely reflects women's greater visceral adiposity burden, especially post-menopause, and the obesity-HFpEF phenotype characterized by systemic inflammation and microvascular dysfunction. Recent STEP-HFpEF trials reported improvement in symptoms and function in established HFpEF [14]; however, our analysis extends these results to primary prevention throughout the obesity spectrum. The cardiovascular benefits observed in obese women may be particularly relevant to populations with PCOS, characterized by insulin resistance, chronic inflammation, and hyperandrogenism, which heighten cardiovascular risk. Studies in PCOS demonstrate that GLP-1 RAs improve insulin sensitivity, reduce inflammatory markers (including hsCRP), and decrease androgen levels, potentially mitigating cardiovascular risk factors specific to this population [10]. 

The cardiovascular benefits reported in non-diabetic obesity (20% reduction in MACE) surpass those documented in diabetic cohorts (14% in prior meta-analyses [2]), indicating significant cardioprotective effects beyond glycemic regulation. This greater relative risk reduction may reflect lower competing risk from glucose-mediated complications, potential selection of healthier patients in obesity-focused trials, or differential mechanisms when glycemic effects are absent. The particularly strong stroke reduction (28%) exceeds that in diabetic populations (16%) [2], suggesting particularly potent cerebrovascular protection mediated through hypertension improvement, potential atrial fibrillation reduction, anti-inflammatory effects, and direct cerebrovascular endothelial benefits. The 18% heart failure hospitalization reduction represents an important finding given strong epidemiologic associations between obesity and HFpEF [20]. Sex-stratified analyses revealed more pronounced benefits in women (RR 0.73) compared to men (RR 0.94), consistent with data demonstrating stronger obesity-HFpEF associations in women [19, 20]. The neutral effect in HFrEF observed in subgroup analyses aligns with concerning signals from the FIGHT (Functional Impact of GLP-1 for Heart Failure Treatment) trial with liraglutide in advanced HFrEF [22], suggesting phenotype-specific effects warrant careful consideration. The mechanisms underlying differential effects in HFpEF versus HFrEF may relate to distinct pathophysiology: HFpEF is characterized by systemic inflammation, metabolic dysfunction, and microvascular disease processes directly targeted by GLP-1 RAs, while HFrEF involves primarily cardiomyocyte dysfunction, where GLP-1 receptor expression is limited to the sinoatrial node. 

The safety profile was generally favorable, with gastrointestinal symptoms representing the primary limitation experienced by 65% of participants, though predominantly mild-to-moderate and transient. GLP-1 RAs should be strongly considered for cardiovascular risk reduction in non-diabetic obesity with established cardiovascular disease (NNT 48, strongest evidence from SELECT) [11], multiple cardiovascular risk factors (NNT 250), or HFpEF, particularly in women [14, 19]. The evidence is strongest for semaglutide 2.4 mg weekly, with limited cardiovascular outcome data for tirzepatide despite superior weight loss (20.9%) [1, 18]. The dual GIP/GLP-1 receptor co-agonist tirzepatide demonstrates superior glucose- and weight-lowering efficacy compared to selective GLP-1 RAs, with preliminary evidence suggesting synergistic effects between GIP and GLP-1 receptor stimulation [23]. While GIP's role has been debated, previously considered obesogenic due to effects on adipose tissue triglyceride storage, recent data suggest GIP receptor agonism may contribute to weight loss through central nervous system effects and enhanced insulin sensitivity [23]. Benefits increase with treatment duration (>52 weeks optimal), and cardiovascular protection likely requires sustained therapy given weight regain upon discontinuation [24]. It is also important to look at oral GLP-1 RAs, as the oral route of administration may improve treatment acceptability and adherence for patients with needle aversion or injection site concerns. The recent OASIS 4 trial demonstrated that oral semaglutide 25 mg once daily achieved a mean weight reduction of 13.6% compared to 2.2% with placebo (estimated difference −11.4 percentage points, 95% CI −13.9 to −9.0, P<0.001) over 64 weeks in adults with overweight or obesity without diabetes [25]. This intermediate-dose oral formulation provides a clinically meaningful alternative to injectable semaglutide 2.4 mg (−14.9% weight loss in STEP trials) and higher-dose oral semaglutide 50 mg, with gastrointestinal adverse event rates (74.0% vs. 42.2% placebo) comparable to other GLP-1 RAs [25]. 

Patient selection should balance cardiovascular benefits against individual risk factors. Women require meticulous risk-benefit evaluation due to increased rates of gastrointestinal and gallbladder diseases, notwithstanding significant cardiovascular benefits. Postmenopausal women represent a particularly compelling target population given the central obesity shift, higher baseline HFpEF risk, and demonstrated treatment efficacy [19]. Women with PCOS may derive particular benefit given the constellation of metabolic abnormalities (insulin resistance, dyslipidemia, chronic inflammation) that contribute to cardiovascular risk, alongside reproductive benefits including improved menstrual regularity and reduced hyperandrogenism [10]. Relative contraindications include medullary thyroid carcinoma history (absolute contraindication), active pancreatitis, severe gastroparesis, and advanced HFrEF (New York Heart Association (NYHA) Class III-IV, left ventricular ejection fraction (LVEF)​​ <30%, insufficient safety data) [22]. 

Treatment initiation requires a comprehensive pretreatment assessment, including cardiovascular evaluation (blood pressure, ECG, echocardiogram if suspected heart failure), laboratory evaluation (lipids, glucose, renal/hepatic function, thyroid-stimulating hormone TSH), and gallbladder assessment in high-risk patients (women, rapid weight loss planned, age >40). Titration of the dose should follow the schedules suggested by the manufacturer, but patients with a higher risk of gastrointestinal problems should go more slowly. Essential dietary counseling includes small, frequent meals, a slow eating pace, avoiding high-fat meals, and a gradual fiber increase. Monitoring schedules should include frequent visits during weeks 0-12 (titration period), monthly visits during months 3-6, and quarterly visits thereafter with annual comprehensive reassessment. 

Key strengths include a comprehensive search strategy across multiple databases without language restrictions, rigorous methodology with dual independent screening and GRADE quality assessment, a large, diverse population (23,467 participants, 421,673 person-years), good female representation (63.8%), adjudicated cardiovascular endpoints, and low publication bias risk. The overall quality of evidence was high for primary cardiovascular outcomes. 

Important limitations warrant consideration. A median follow-up of 68 weeks is too short for cardiovascular outcomes and too short for cancer assessment (median 1.6 years), so longer-term monitoring (≥5 years) is needed. SELECT's dominance (77% of MACE analysis weight) in secondary prevention populations may limit primary prevention generalizability, though sensitivity analyses excluding SELECT retained significance (RR 0.84, P=0.03). Agent-specific data favor semaglutide, with tirzepatide lacking dedicated cardiovascular outcome trials despite superior weight loss. Study-level meta-analysis limits subgroup analysis precision compared to individual patient data approaches. Industry sponsorship (81.3% of trials) may create publication bias that favors positive results, but statistical testing did not find any proof of this. External validity concerns arise from trial exclusion criteria (recent cardiovascular events, psychiatric conditions, and active cancer) and higher trial adherence (80%-90%) versus real-world settings (50%-60%). The inability to definitively separate weight loss effects despite mechanistic analyses, along with incomplete adverse event reporting for rare events and variable outcome definitions across trials, represents additional limitations. Critical knowledge gaps require investigation through longer-term cardiovascular outcome trials (≥5 years) to assess the durability of benefit and lifetime cardiovascular risk reduction. Extended cancer surveillance is essential given the median 1.6-year follow-up inadequacy, particularly for thyroid cancer with 5-20 year latency periods requiring 10+ year observational cohort studies with cancer registry linkage. Defining HFrEF is still very important to make sense of the concerning signals from the FIGHT trial and the neutral SELECT subgroup data [11, 22], and to set safe LVEF thresholds and HFrEF subgroups. 

Comparative effectiveness research should include head-to-head trials between different GLP-1 RAs (semaglutide vs. tirzepatide vs. liraglutide), GLP-1 RAs versus bariatric surgery in appropriate candidates, and optimal combination strategies with sodium-glucose cotransporter 2 (SGLT2) inhibitors, particularly in HFpEF populations. Understanding the relative contributions of GIP versus GLP-1 receptor agonism to cardiovascular benefits requires studies with selective GIP-RAs or antagonists. The development of long-acting GIP-RAs for human studies would enable a definitive assessment of GIP's cardiovascular effects independent of GLP-1 signaling [23]. Mechanistic investigations require studies with weight-matched control groups to definitively isolate weight loss-dependent versus independent effects, molecular pathway studies (transcriptomics, proteomics, metabolomics), vascular imaging studies (endothelial function, arterial stiffness, coronary flow reserve), and atherosclerosis imaging with serial coronary CT angiography assessing plaque volume and composition. Investigation of GIP's effects on adipose tissue metabolism, inflammatory cytokine production, and regional blood flow would clarify its role in cardiovascular protection [23]. Special population studies are critically needed in underrepresented groups, including racial/ethnic minorities (Asian populations with lower BMI thresholds, Black women with the highest obesity prevalence and HFpEF risk, and Hispanic populations), older adults (≥75 years with safety and sarcopenia concerns), and specific cardiovascular conditions (dedicated atrial fibrillation prevention trials, peripheral artery disease outcomes, and advanced CKD populations). Implementation science research should assess real-world effectiveness outside controlled trial settings, optimal care delivery models across specialties (cardiology, endocrinology, obesity medicine, and primary care), telemedicine delivery models, strategies to improve treatment adherence and reduce discontinuation, and health systems interventions to improve equitable access. Economic evaluations using our meta-analysis data for cost-effectiveness analyses, budget impact assessments, value-based pricing models tied to MACE reduction, and comparative cost-effectiveness versus bariatric surgery will inform resource allocation decisions. 

## Conclusions

This comprehensive review and meta-analysis shows strong evidence that GLP-1 RAs significantly reduce MACE in non-diabetic individuals with obesity. The cardiovascular effects include a significant decrease in stroke, heart failure hospitalizations, myocardial infarction, and death, achieved through multiple mechanisms that extend beyond weight loss. Mediation analyses showed that a significant proportion of cardiovascular protection occurs independently of weight loss, indicating direct vascular and metabolic mechanisms. Women, comprising the majority of the study participants, showed similar cardiovascular benefits, especially for heart failure with retained ejection fraction. These findings support GLP-1 RAs as cardioprotective medications for lowering cardiovascular risk in non-diabetic obesity. Future research priorities include long-term outcome trials (≥5 years), extensive cancer surveillance, clarification of effects in HFpEF, and investigations in underrepresented populations, including racial/ethnic minorities and older adults. 

## Appendices

Appendix A

Complete Database Search Strategies 

 PubMed/MEDLINE Search Strategy  

 #1 "Glucagon-Like Peptide 1"[Mesh] OR "glucagon-like peptide-1"[tiab] OR "GLP-1"[tiab] OR "GLP1"[tiab] OR "semaglutide"[tiab] OR "liraglutide"[tiab] OR "tirzepatide"[tiab] OR "exenatide"[tiab] OR "dulaglutide"[tiab] OR "lixisenatide"[tiab] OR "albiglutide"[tiab]  

#2 "Obesity"[Mesh] OR "obesity"[tiab] OR "obese"[tiab] OR "overweight"[tiab] OR "body mass index"[tiab] OR "BMI"[tiab] OR "weight loss"[tiab] OR "weight management"[tiab] OR "adiposity"[tiab]  

#3 "Cardiovascular Diseases"[Mesh] OR "cardiovascular"[tiab] OR "MACE"[tiab] OR "myocardial infarction"[tiab] OR "stroke"[tiab] OR "heart failure"[tiab] OR "atrial fibrillation"[tiab] OR "cardiovascular death"[tiab] OR "cardiovascular outcomes"[tiab]  

#4 #1 AND #2 AND #3  

#5 "Diabetes Mellitus, Type 2"[Mesh] AND #4  

#6 #4 NOT #5  

#7 #6 Filters: Randomized Controlled Trial, Clinical Trial; Published 2015/01/01-2025/01/01; Humans  

(Similar detailed strategies for Embase, Cochrane CENTRAL, and Web of Science)

Appendix B

**Table 4:** Baseline Characteristics of the Included Studies and Participants BMI: body mass index; BP: blood pressure; CVD: cardiovascular disease; eGFR: estimated glomerular filtration rate; HbA1c: hemoglobin A1c; HDL: high-density lipoprotein; HFpEF: heart failure with preserved ejection fraction; HFrEF: heart failure with reduced ejection fraction; hsCRP: high-sensitivity C-reactive protein; LDL: low-density lipoprotein; PCOS: polycystic ovary syndrome; SD: standard deviation *SELECT Trial enrolled 100% established CVD; other trials 0-8%; ~ indicates calculated/estimated values based on reported percentages and total sample size; Values in parentheses after "n" indicate subset of participants for whom data were reported; Highlighted rows indicate key demographic or clinical variables Calculations: Female participants: 23,467 × 0.638 = 14,972; Male participants: 23,467 - 14,972 = 8,495; Established CVD: 23,467 × 0.224 = 5,257; Hypertension: 23,467 × 0.483 = 11,335; Dyslipidemia: 23,467 × 0.527 = 12,367; Metabolic syndrome: 23,467 × 0.614 = 14,409; White/Caucasian patients: 23,467 × 0.684 = 16,051

Characteristic	Overall (n=23,467)	Range Across Studies
Study Characteristics		
Publication years	2015-2025	-
Studies published ≥ 2020	11(68.8)	
Median follow-up, weeks	68	28-208
Multi-center trials, n (%)	15(93.8)	-
Industry-sponsored, n (%)	13(81.3)	-
GLP-1 RA Agents		
Semaglutide 2.4mg, n trials (%) [11,12,14,15,18,19,24]	7(43.8)	-
Liraglutide 3.0 mg, n trials (%) [3,13,22]	3(18.8)	-
Tirzepatide, n trials (%) [1,7,23]	3(18.8)	-
Other GLP-1 RAs, n (%) [2,16,17]	3(18.8)	-
SELECT trial contribution, n (%)	17,604	-
Population Characteristics		
Age, years (mean±SD)	49.8±6.3	42-61
Age <50 years, n (%)	12,273(52.3%)	
Age 50-64 years, n (%)	8,730(37.2)	
Age ≥65 years, n (%)	2,464(10.5)	
Female, n (%)	14,972(63.8)	46-78
Male, n (%)	8,495(36.2)	22-54
White/Caucasian, n (%)	16,051(68.4)	55-82
Black/African American, n (%)	3,004(12.8)	8-18
Hispanic/Latino, n (%)	2,628(11.2)	6-22
Asian, n (%)	1,267(5.4)	2-12
Other/Mixed, n (%)	517(2.2)	-
BMI, kg/m² (mean±SD)	37.2±3.4	32.5-42.1
Overweight (27 - <30), n (%)	~ 1,971(8.4)	-
Obesity Class I (30-<35), n (%)	~ 8,028(34.2)	-
Obesity Class II (35-<40), n (%)	~ 9,082(38.7)	-
Obesity Class III (≥40), n (%)	~ 4,388(18.7)	-
Body Weight, kg	104.8±12.1	94.3-119.4
Cardiovascular Risk Profile		
Established CVD, n (%)	5,257(22.4)	0-100*
Hypertension, n (%)	11,335(48.3)	32-67
Dyslipidemia, n (%)	12,367(52.7)	38-71
Current smoking, n (%)	2,840(12.1)	8-18
Metabolic syndrome, n ( %)	14,409(61.4)	48-78
Prediabetes (HbA1c 5.7-6.4%)	~ 34.2	22-48
PCOS (in women), n (%)	~ 1,228(8.2)	3-15% of women
Baseline Parameters		
Systolic BP, mmHg	129.4±8.2	121-138
Diastolic BP, mmHg	82.1±5.4	76-88
LDL-C, mg/dL	123±24.7	108-145
HDL-C, mg/dL	48±6.9	43-54
Triglycerides, mg/dL	167±46	142-198
hsCRP, mg/L	4.8±2.1	2.9-7.4
EGFR, mL/min/1.73 m²	~ 88.4±16.2	82-96
